# Housing affordability and health in people with disability: A scoping review

**DOI:** 10.1093/epirev/mxag004

**Published:** 2026-03-09

**Authors:** Kate Mason, Tanya Durrand, Glenda M Bishop, Alex Sully, Zoe Aitken

**Affiliations:** Healthy Housing Unit, Centre for Health Policy, Melbourne School of Population and Global Health, University of Melbourne, Carlton, VIC, Australia; Disability and Health Unit, Centre for Health Policy, Melbourne School of Population and Global Health, University of Melbourne, Carlton, VIC, Australia; Disability and Health Unit, Centre for Health Policy, Melbourne School of Population and Global Health, University of Melbourne, Carlton, VIC, Australia; Disability and Health Unit, Centre for Health Policy, Melbourne School of Population and Global Health, University of Melbourne, Carlton, VIC, Australia; Disability and Health Unit, Centre for Health Policy, Melbourne School of Population and Global Health, University of Melbourne, Carlton, VIC, Australia

**Keywords:** disability, disabled persons, health inequities, housing, mental health, social determinants of health

## Abstract

Persistent inequities in the social determinants of health contribute to poor health outcomes among people with disability. People with disability are more likely to live in unaffordable housing, which is associated with increased risk of chronic conditions and poor mental health. However, there is limited research on how housing affordability affects health in this priority population. We undertook a scoping review to evaluate existing evidence on the health impacts of living in unaffordable housing for people with disability. Peer-reviewed literature published in English between 2004 and 2024 was considered. The MEDLINE, SocIndex, and Web of Science databases were searched to identify studies examining possible links between housing affordability and any health outcome in a population of people with disability. Two independent reviewers performed study screening and data extraction. Of the 1386 studies identified initially, 3 met the eligibility criteria. The included studies focused on individuals with acquired, psychiatric, or military-related disability. Each study provided evidence suggesting unaffordable housing negatively affected mental health in people with disability. Substantial methodological and conceptual heterogeneity limited direct comparison or synthesis. This review highlights a critical knowledge gap in the relationship between housing affordability and health for people with disability, limiting the development of evidence-informed policy and intervention. People with disability have a fundamental right to access affordable, appropriate housing, as well as the right to the highest attainable standard of health. High-quality, targeted research using consistent definitions of disability and housing affordability, as well as validated health outcome measures, is needed to inform future policy.

## Introduction

The United Nations Convention on the Rights of Persons with Disabilities states that people with disability are entitled to the fundamental human right to access adequate, affordable housing.^1^ Housing is a social, cultural, environmental, and economic determinant of health that is essential to provide security and stability, and to meet the basic needs of individuals and families.^2^^,^^3^ A substantial body of evidence links housing to health and health equity, dating back to the 19th century discovery that overcrowding and poor ventilation promote the spread of communicable diseases such as tuberculosis and cholera.^4^ Much of the existing research has focused on the impact of specific physical housing-related exposures (eg, mold and damp, overcrowding) on specific health outcomes (eg, respiratory health). More recently, because housing affordability has become a pressing housing concern in many higher-income countries^5^—exacerbated by the erosion of social housing provision in recent decades—attention on the health impacts of living in unaffordable housing has grown.

Fundamentally, housing affordability refers to the relationship between household income and housing costs, and relates to the notion that access to adequate housing should not compromise well-being or the ability to afford other essential expenses.^6^ Not only can unaffordable housing directly affect, for example, mental health, but because high housing costs often push lower-income households into housing that is of poorer quality and less well located, as well as cause individuals to constrain their spending on other essentials such as health care, housing affordability also has plausible indirect pathways to a broader range of health outcomes.^3^^,^^7^

People with disability are more likely to live in unaffordable housing and consistently experience poorer housing outcomes than are people without disability.^8^^,^^9^ On average, people with disability also experience poorer mental and physical health than people without disability.^10^^,^^11^ Housing affordability may be a driver of these health inequalities; evidence suggests the health inequalities experienced by people with disability are largely driven by social determinants of health such as socioeconomic status and living conditions, and structural barriers such as stigma and discrimination.^12^ Partly due to often having more health-related expenses, people with disability also often have higher living costs than those without disability,^13^ meaning the association between living in unaffordable housing and negative health outcomes might be especially pronounced in this population if high housing costs limit capacity to afford essential health-related expenses.

The term *housing affordability* is widely used in academic, policy, and public discourse in many countries, though its precise definition varies in different contexts.^14^ One conventional definition of affordability is the housing cost-to-income ratio, which defines housing as unaffordable when the proportion of household income allocated to rent or mortgage payments exceeds a particular threshold, which is typically—though somewhat arbitrarily—set at 30%.^3^ However, this definition does not account for higher-income households potentially choosing to spend more than 30% of their income on housing, without compromising their ability to afford other essentials. Recognizing this, in countries such as Australia, the “30/40” indicator is used in research and policy, classifying households as in “housing stress” if they spend more than 30% of household income on housing costs while also being in the lowest 40% of the national income distribution.^15^ Other definitions of housing affordability include the residual income approach, whereby housing is deemed unaffordable if a household has insufficient income for other essential needs, such as food and transport, after paying for housing costs.^16^ Usually, housing costs are defined as rent and mortgage payments, though some definitions cover a broader set of housing-related costs including rates and utility bills; others propose recognizing costs incurred as a result of home location (eg, commuting expenses).^17^

A growing body of evidence demonstrates direct and indirect links between housing affordability and health.^2^ Associations have been demonstrated between living in unaffordable housing and an increased risk of chronic conditions such as hypertension and arthritis,^18^ as well as poor mental health and self-rated health.^18^^-^^21^ Some adverse health outcomes are likely to arise directly from exposure to unaffordable housing costs, while others may result from a reduced ability to afford health care^19^ or food.^22^ Housing affordability also has implications for both housing quality and location, and there is increasing recognition of the wider impact of this on health outcomes.^17^ Constrained by affordability, low-income households are more likely to live in poorer quality housing that is less energy efficient, requiring trade-offs between meeting high utility bills and other essential costs.^23^^,^^24^ Furthermore, in many cities, housing that is affordable for low-income households is often located in outer-urban areas that are under-serviced and lack adequate access to transport; the resultant spatial dislocation can negatively affect health through social isolation and reduced access to essential services, and high-transport costs that divert money away from other essential needs.^17^^,^^25^

In recent decades, increasing housing costs have been outstripping wage growth in many high-income countries, leading to escalating housing affordability challenges.^5^^,^^26^ Understanding the health implications of living in unaffordable housing, therefore, is more critical than ever. Although existing evidence goes some way to demonstrating the adverse health outcomes linked to unaffordable housing in the population at large, there is a significant lack of research focused on population subgroups most at risk for these effects. People with disability were the largest minority group worldwide, constituting an estimated 16% of the global population, or 1.38 million people, in 2022,^12^ but despite the increased likelihood of exposure to unaffordable housing among this population, we are unaware of any published attempt to synthesize existing evidence about the impacts of unaffordable housing on the health of people with disability. This is particularly important given not only the size of this minority group but also the diversity of experiences within it. This diversity occurs because disability, as conceptualized by the International Classification of Functioning, Disability, and Health (ICF) framework, is a complex biopsychosocial phenomenon that results from an interaction between a person’s health condition and other personal and environmental factors, restricting the person’s capacity to participate effectively in society.^27^

Our research question, therefore, was: What is the impact of living in unaffordable housing on health outcomes in people with disability? Answering this question has the potential to inform future policy and interventions to improve housing affordability and health outcomes in people with disability. A preliminary search of the MEDLINE, the Cochrane Database of Systematic Reviews, and JBI Evidence Synthesis databases was conducted and no current or underway systematic reviews or scoping reviews on the topic were identified. Because this is a broad topic with an expected lack of research, we deemed a scoping review to be the most appropriate method for mapping the available evidence and identifying gaps in knowledge.^28^

## Methods

Our scoping review followed the established scoping review methodology guidelines outlined by JBI.^29^ This included use of the Preferred Reporting Items for Systematic Reviews and Meta-Analyses (PRISMA)^30^ extension for scoping reviews, ensuring comprehensive reporting of findings. A preliminary review framework and objectives were established and adapted as needed throughout the review process. A review protocol was not published.

We conceptualize disability in line with the ICF framework, published in 2001 and endorsed by the World Health Organization, which defines disability as a long-term health condition or impairment lasting longer than 6 months and results in difficulties—described as activity limitations or participation restrictions—functioning in daily life.^27^ This framework integrated the existing social and medical models of disability to form the current biopsychosocial model. From this perspective, an individual’s level of functioning is dynamic rather than static and influenced by health, environmental, and personal factors. Furthermore, a broad definition of “disability” is used to encompass functioning and participation across multiple life domains. This means disability is understood not simply as the presence of an underlying health condition but as the extent to which it affects an individual’s capacity to undertake usual activities of daily life.

### Eligibility criteria

The relationship among housing costs, disability, and health is complex. Although having a disability can, through various pathways (eg, increased health care costs or limited income), increase an individual’s risk of housing affordability stress, in this review we focus on identifying studies that examined the influence of housing affordability on the health of people with disability. We used the following inclusion criteria to identify studies for our review: studies that (1) included a clearly defined population of people with disability, (2) examined the concept of housing affordability (using any definition) as an exposure that could influence health, (3) reported on at least 1 health outcome, and (4) were published in a peer-reviewed journal in English between January 2004 and May 2024. A broad range of study types, including quantitative and qualitative primary research, systematic reviews, and scoping reviews, were considered for inclusion. Commentaries, frameworks, and editorials were excluded. We limited our scope to studies conducted in the past 20 years based on the introduction of the ICF framework in the early 2000s.

Due to the expected paucity of research on this topic, our definition of a health outcome was broad and included social determinants of health. Eligible social determinants of health were defined according to the World Health Organization’s examples of social determinants of health that can positively and negatively influence health equity, such as food insecurity, social inclusion, and access to affordable health services.^31^ Furthermore, studies reporting on an eligible social determinant of health needed to explicitly frame this in the context of health—which we defined as use of the words “health” or “well-being” in the title or abstract, or publication in a health-related journal. Studies using the term “housing insecurity” rather than “housing affordability” in the title or abstract were also considered because housing insecurity can encapsulate housing affordability, and this may not be evident until the full text is reviewed.

Articles were excluded if they (1) involved a population of people with a health condition but did not include use of disability language (ie, using only the name of a diagnosis without the word “disability” or similar), (2) focused on disability as an outcome, or (3) focused on homelessness (although homelessness is often connected to housing affordability, it is a specific and complex experience beyond the scope of this review.)

### Search strategy

A limited search of MEDLINE was undertaken to test appropriate terminology to identify articles on the review topic. An initial search strategy was then developed using keywords in the titles and abstracts of relevant articles and the associated index terms. Development of the final search strategy, selection of appropriate databases, and adaptation of the search for each database were undertaken with input from an experienced health sciences and medical research librarian (see Appendix S1 for details of the final search strategy). The final search was executed in MEDLINE and SocIndex on May 6, 2024, and in Web of Science on May 7, 2024. The reference list of each included study was subsequently screened to identify additional eligible studies. We did not search the gray literature, because our focus was on peer-reviewed evidence.

### Study selection

Identified citations from each database were imported into Covidence, which is a web-based screening and data extraction program (Veritas Health Innovation, Melbourne, Australia; www.covidence.org). After automated removal of duplicates, all titles and abstracts were screened independently by 2 reviewers (T.D. and A.S.) for assessment against the eligibility criteria, and undetected duplicates were marked manually. Full-text PDFs were retrieved for all citations screened as eligible at this stage, and a list of reasons for full-text exclusion was created based on the eligibility criteria. Each full-text study was assessed in detail against the eligibility criteria by 2 independent reviewers (T.D. and A.S.) and reasons for exclusion recorded, with the final decisions also verified by the entire review team. At each stage of the screening process, conflicts were discussed among the entire research team and resolved through consensus.

### Data extraction

A clear and comprehensive data extraction template was created based on JBI guidelines.^29^ Two reviewers (T.D. and A.S.) independently extracted the relevant information from the included studies. Extracted data included study identifiers and location, methodology, population and sample size, details of the conceptualization of disability and housing affordability, health outcome(s) evaluated, key findings, and policy recommendations. The extracted data were verified by the entire research team, who read the included studies in full before meeting to resolve small inconsistencies in the extracted data.

### Data synthesis

A narrative synthesis approach was used to analyze the extracted data. We adopted a descriptive and integrative approach to narrative synthesis to allow for a summary and explanation of the findings across the studies in relation to the review question. This method of data analysis was chosen because the heterogeneity of the included studies precluded a thematic analysis.^32^

## Results

### Included studies

**Figure 1:**
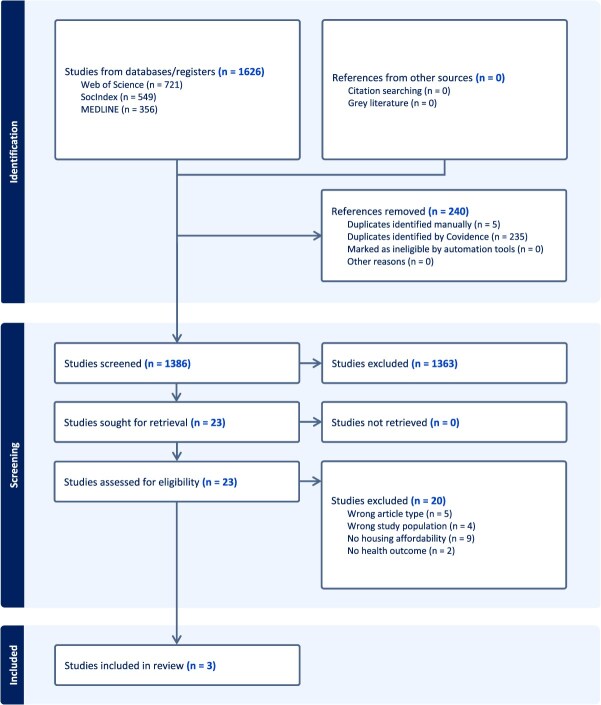
Preferred Reporting Items for Systematic Reviews and Meta-Analyses (PRISMA) flow diagram illustrating literature search and screening.

Our search retrieved 1626 articles, of which 240 were duplicates. Of the 1386 unique records we screened, 1363 were excluded upon review of the title and abstract, because they were not directly relevant to the review (ie, there was no population of people with disability, no mention of housing affordability, and/or no health outcome). Of the remaining 23 full-text articles considered for inclusion, 20 were excluded after a detailed assessment against the eligibility criteria. Reasons for full-text exclusion were wrong article type, wrong population, did not examine housing affordability, or had no health outcome. Many studies met more than 1 of these criteria for full-text exclusion. This left 3 studies for inclusion in our review. The PRISMA flow diagram in Figure 1 depicts details of this process.

### Study characteristics

The 3 included studies were published in 2008, 2016, and 2019. Two studies were conducted in Australia,^33^^,^^34^ and 1 in the United States.^35^ The included studies involved 3 broad categories of disability: acquired disability,^33^ psychiatric disability,^34^ and military-related disability.^35^ Of the 3 studies, 2 used a longitudinal design—1 quantitative and 1 mixed-methods—and the third was a qualitative cross-sectional study. Kavanagh et al.^33^ used fixed-effects longitudinal regression analysis to investigate if housing tenure and affordability modified the relationship between disability acquisition and mental health. Their study population, consisting of 1913 people who acquired a disability in adulthood, was drawn from 12 waves of annual data collected between 2001 and 2012 as part of the Household, Income, and Labour Dynamics in Australia (HILDA) Survey, a nationally representative annual survey of Australian adults. Muir et al.^34^ conducted a longitudinal mixed-methods evaluation of the New South Wales (NSW) Mental Health Housing and Accommodation Support Initiative (HASI), which is a supported social housing program for clients with high-level psychiatric disability. Using a sample of 113 clients, the study assessed the program’s impact across multiple outcomes, including health outcomes, using data collected from 633 qualitative interviews and surveys with clients and key stakeholders, along with quantitative data, both gathered at 6-month intervals over 2 years. Finally, Semeah et al.^35^ conducted a descriptive, qualitative study to explore the facilitators and barriers faced by 39 veterans with military-related disabilities in finding and maintaining suitable rental housing in the United States. These facilitators and barriers were identified through a 48-item questionnaire including open-ended questions, with thematic analysis used to identify key themes. A full description of the included studies is available in Table 1.

**Table 1:** Description of included studies in scoping review of housing affordability and health in people with disability, 2004-2024.

	**Objective**	**Design**	**Disability population**	**Affordability**	**Outcome measures**	**Key findings** ^**a**^
Kavanagh et al.^33^ 2016 Australia	To investigate if housing tenure and affordability modify the effect of disability acquisition on mental health	Longitudinal fixed-effects regression analysis of 12 waves of HILDA data (2001-2012)	**Type:** disability acquired in adulthood (*n* = 1913) **Model:** ICF **Definition:** participants asked if they had an “impairment, long-term health condition or disability which restricts their everyday activities that had lasted, or was likely to last, for a period of 6 months or more.”	**Definition:** households with a disposable income in the lowest 40% of the annual national distribution, with housing payments > 30% of gross household income	**Mental health:** self-reported mental health measured using the MCS score of the SF-36 health questionnaire	People who acquired a disability and lived in unaffordable housing had a greater decline in MCS scores (4.2 points) than those in affordable housing (1.7 points)
Muir et al.^34^ 2008, Australia	To evaluate the effectiveness of HASI in improving community participation, tenancy sustainability, and mental health	Longitudinal mixed-methods evaluation, with data collected at 6 mo intervals over 2 y	**Type:** “high-level” psychiatric disability (*n* = 113) **Model:** not specified **Definition:** not specified	**Definition:** not specified: “clients were provided with affordable, secure social housing”	**Mental health (*n* = 67):** NSW Health hospital data, and interviews and surveys **Physical health (*n* = 55):** Interviews and surveys **Well-being (n = 55):** PWI	**Mental health:** “improved for most clients” based on decreased psychiatric hospital admissions for 84% of clients, and interview/survey results **Physical health:** “improved in 60%” **Well-being:** decreased across all PWI domains
Semeah et al.^35^ 2019, United States	To identify facilitators and barriers to finding and maintaining suitable rental housing for veterans with military-related disability	Qualitative descriptive study with thematic analysis	**Type:** military-related disability (*n* = 39) **Model:** biopsycho-ecological **Definition:** “a physical and/or psychological condition, disease, or injury sustained during duty”	**Definition:** not specified (but emerged as a theme in the analysis)	48-item questionnaire, including open-ended questions	Veterans reported that affordable housing was often in unsafe neighborhoods, which can influence substance abuse recovery and social isolation.

a HASI, Housing and Accommodation Support Initiative; HILDA, Household, Income, and Labour Dynamics in Australia; ICF, International Classification of Functioning, Disability, and Health; MCS, Mental Component Summary; NSW, New South Wales; PWI, Personal Wellbeing Index. We only report on findings related to our research question.

### Conceptualization of disability populations

Kavanagh et al.^33^ focused on individuals who acquired a disability in adulthood; the researchers used a definition of disability derived from the ICF model of disability. Their definition included any disability, impairment, or long-term condition causing a restriction in daily activities lasting at least 6 months. Participants were characterized as having an acquired disability if they did not report a disability for at least 2 consecutive waves followed by at least 2 consecutive waves with a reported disability. Muir et al.^34^ did not specify what constitutes a “high level of psychiatric disability” in the clients participating in their program evaluation, and they did not mention the use of an overarching model of disability. However, they provided a breakdown of the primary diagnoses in the study population: 74.3% of participants had schizophrenia, and others were diagnosed with schizoaffective disorder (11.9%), bipolar disorder (2.8%), depression (1.8%), or other conditions (9.2%). Furthermore, almost 65% of clients had a secondary diagnosis of a past (45.9%) or current (29.6%) substance use disorder, a physical disability (17.3%), an intellectual or cognitive disability (31.6%), or a secondary mental illness (11.2%). Finally, Semeah et al.,^35^ focused on veterans with a military-related physical and/or psychological disability, and they conceptualized disability using the biopsycho-ecological model. This model defines disability as “arising from interactions between mental and physical characteristics and the physical and social environments.”^36^ Participants were asked if they had a physical and/or psychological injury, disease, or condition as a result of their military duty. Many veterans had more than 1 type of disability, categorized by the authors as musculoskeletal (51%), mental health (74%), trauma related (21%), neurological (51%), and other (13%).^35^

### Housing affordability measures

In the study by Kavanagh et al.,^33^ unaffordable housing was defined as households with a disposable income in the lowest 40% of the annual national distribution, with housing payments greater than 30% of household gross income. This definition is in line with the aforementioned 30/40 approach that is frequently used to define housing affordability in policy and research. Muir et al.^34^ did not define housing affordability, because their study did not focus on this directly. Instead, they stated the HASI program evaluated in the study involved the provision of affordable housing as 1 component of the program providing clinical mental health case management and support to develop skills and increase community participation. Semeah et al.^35^ also did not define housing affordability explicitly; rather, it emerged as a key barrier to finding and maintaining suitable rental housing for participants with military-related disability. The authors also characterized some participants as being cost burdened, with moderately cost burdened defined as spending 30% to 50% of their income on housing costs and severely cost burdened defined as spending greater than 50% of their income on this category.^35^

### Health outcome measures

Across the included studies, a range of health outcomes was covered. Kavanagh et al.^33^ measured mental health using a Mental Component Summary (MCS) score, derived from a widely used Short Form 36 (SF-36) survey, a self-completed questionnaire comprising 36 questions relating to health status. The MCS score is derived by combining responses from multiple questions, heavily weighted toward the mental health components of the SF-36, and is designed to capture mental health and well-being, rather than clinical mental health conditions. Muir et al.^34^ measured 3 health outcomes. First, they evaluated mental health using NSW Health hospitalization data and semi-structured interviews and surveys. Second, client well-being was measured using a modified Personal Wellbeing Index (PWI)—a quantitative measure of quality of life across 7 domains, including health, with 2 additional domains for satisfaction with mental and physical health added by the authors. Third, physical health was evaluated using surveys and semi-structured interviews. Semeah et al.^35^ did not specifically evaluate health as a primary outcome; however, mental health outcomes emerged in the responses to the open-ended questions.

### Key results from each study

#### Kavanagh et al.

Kavanagh et al.^33^ found statistical evidence that housing affordability modified the effect of disability acquisition on mental health. The analysis demonstrated that people who had acquired disability and lived in unaffordable housing experienced a 4.2 point decrease in MCS scores (95% CI, 5.2-1.4), compared with a 1.7 point decrease for those who acquired a disability and lived in affordable housing (95% CI, 2.1-1.3). The authors concluded that the magnitude of the mental health effect of acquiring a disability was larger for people living in unaffordable housing than it was for people living in affordable housing.

#### Muir et al.

Based on the 67 clients who participated in the entire evaluation and had linked psychiatric hospital admission data, Muir et al.^34^ reported that “overall mental health increased for most clients.” Using NSW Health hospitalization data from 5 financial years—before and during the program—the authors found that 84% of clients were hospitalized for less time compared with the years immediately before the program, and that average time spent in hospital decreased from 24.3% before the program to 4.6% during the program.

Additionally, Muir et al. reported physical health improvement in 60% of the 55 clients who participated in the entire evaluation and had complete survey data, although approximately 1 in 5 clients reported declining physical health in the final phase of the evaluation.^34^ The authors noted that many clients of the program had a range of chronic conditions and were more than twice as likely as the general Australian adult population to describe their general health as fair or poor. In contrast, self-reported well-being decreased across all PWI domains between the first and final phase of the program evaluation in the 55 clients who participated in the entire evaluation. The mean decreases in the PWI domains included general health (from 56.0 to 49.2), mental health (from 66.7 to 58.2), and physical health (from 56.9 to 53.4). The authors suggested the overall decrease in mean PWI scores was more likely to be attributed to a change in the clients’ aspirations and expectations after the initial “honeymoon period,” rather than limitations in the HASI program.^34^ They also reported that clients were more satisfied with each domain than not: scores for all domains, barring general health but including physical and mental health, remained above 50 at all follow-up points, despite the overall decrease.

#### Semeah et al.

Through 4 rounds of coding of their questionnaire responses, Semeah et al.^35^ generated 5 themes encapsulating the facilitators and barriers faced by veterans with military-related disabilities in finding and maintaining suitable rental housing: policies, communication, quality neighborhood, lack of quality housing, and reintegration. These themes did not directly focus on health outcomes or housing affordability, although housing affordability was a theme in the first 2 rounds of coding. Within the quality neighborhood theme, the authors reported that a lack of affordable housing within appropriate neighborhoods was a key barrier to finding and maintaining suitable rental housing. Furthermore, some participants reported that affordable housing was often located in unsafe areas with exposure to negative influences, such as drug use and crime, which had impacts on substance abuse recovery. One participant expressed that veterans often experienced depression, which was exacerbated by social isolation and lack of access to community services as a result of living in low-socioeconomic neighborhoods.

### Summary of findings

All 3 studies reported findings related to mental health outcomes, indicative of a detrimental effect of living in unaffordable housing on mental health in people with disability. Kavanagh et al.^33^ found that people who acquired a disability and lived in unaffordable housing had a greater decline in mental health scores compared with those in affordable housing, whereas Muir et al.^34^ found that a program providing supported, affordable housing significantly decreased psychiatric hospital admissions in people with a high level of psychiatric disability. In addition to examining mental health, Muir et al also examined well-being and physical health as additional outcomes, with mixed, and some paradoxical, findings. Semeah et al.^35^ identified a lack of affordable housing in safe, quality neighborhoods, and highlighted the negative impact of this on substance abuse recovery and social isolation, with implications for physical and mental health.

## Discussion

There is increasing recognition of housing affordability as a social determinant of health, with evidence suggesting a detrimental impact of living in unaffordable housing on a range of health outcomes. It is also known that people with disability are more likely to live in unaffordable housing and to face inequitable health outcomes. Despite this, evidence on the relationship between housing affordability and health in this priority population is scarce. In this scoping review, we aimed to identify and describe the available literature on the impact of housing affordability on health outcomes in people with disability.

With only 3 studies meeting the eligibility criteria, and only 1 directly addressing the review question, our findings highlight the scarcity of peer-reviewed research on this topic. This reveals a critical gap in the literature, with important implications for the development of evidence-informed policy and intervention in this space. However, all 3 of the studies we found demonstrated some evidence of a negative impact of living in unaffordable housing on mental health outcomes for people with disability. Although synthesizing the results in a meaningful way was challenging, due to substantial heterogeneity across the key areas of the review question, our review nonetheless provides a starting point for future research and offers valuable insights into the limitations of existing studies to assist in refining future research efforts.

### Heterogeneity between studies

Each study focused on a different population of people with disability (acquired, psychiatric, and military related) using different models of disability (ICF and biopsycho-ecological). One study specified neither a model of disability nor what constituted the “high level” of psychiatric disability that defined their cohort, and it was unclear how the authors conceptualized psychiatric disability. The various types of disability in the included studies reflects the diversity within the broader population of people with disability, and it is critical to represent this in public health research. However, it is difficult to compare findings between a small number of studies that each focus on a specific, differing population. Although the 3 studies all evaluated different disability cohorts and described different models of disability, importantly, they all considered disability not solely based on the presence of a health condition but where there was also an interaction between personal and environmental factors that restricted everyday activities, essentially aligning with the globally accepted biopsychosocial model of disability defined by the ICF.

Conceptualization of housing affordability also varied across the included studies. Kavanagh et al.^33^ were the only authors to conceptually define housing affordability; furthermore, theirs was the only study with housing affordability at the core of its methodology and analysis. Muir et al.^34^ did not explicitly focus on affordability but stated the housing program they evaluated provided affordable housing, and they implied that a lack of affordable housing was a contributor toward the vulnerable housing status of the participants, and therefore that this affected outcomes. In their thematic analysis, Semeah et al.^35^ identified housing affordability as 1 of numerous barriers for participants in obtaining appropriate rental housing. In the latter study, the authors acknowledged that the introduction to their survey stated there was a lack of suitable and affordable housing for people with disability, which may have primed the respondents and introduced bias.

The health outcome measures used also varied significantly across the studies. Although all 3 studies focused on mental health in some way, the outcome measures varied widely in quality. Kavanagh et al.^33^ used a clearly defined, quantitative measure of mental health. Muir et al.^34^ used a clearly defined quantitative measure of well-being, but their measurement of mental health (and physical health) was less well defined. Semeah et al.^35^ did not directly measure a health outcome, though substance abuse recovery and social isolation emerged from the results of a 48-item questionnaire with open-ended questions.

### Synthesis of key findings

Despite the heterogeneity between studies, all studies reported findings on the impact of unaffordable housing on mental health. In their well-defined study. Kavanagh et al.^33^ demonstrated that individuals who acquired a disability and lived in unaffordable housing experienced a substantially larger decline in mental health scores compared with those who were living in affordable housing. Muir et al.^34^ found a significant decrease in psychiatric hospitalizations for individuals with high-level psychiatric disability who participated in a program providing supported, affordable housing. That decrease in hospitalizations reportedly reflected an improvement in mental health for most clients, though it was an indirect measure of mental health, and the multifaceted nature of the intervention made it impossible to isolate the impact of housing affordability. The positive mental health findings of Muir et al were contradicted by the decrease in all domains of their well-being measure.^34^ Finally, Semeah et al.^35^ reported that a lack of affordable housing in safe, quality neighborhoods negatively affected substance abuse recovery and social isolation in veterans with military-related disability. Although those outcomes were related to mental health, the study was only marginally focused on those outcomes.

Due to the high level of heterogeneity across the key areas of the review question, synthesizing the results in a systematic way was not feasible. Furthermore, the utility of the results from 2 of the 3 studies was significantly constrained by their indirect relevance to our review question, particularly due to the unclear impact of housing affordability and the secondary or poorly defined outcome measures. One study provided clear empirical evidence that unaffordable housing was associated with a significantly larger decline in mental health scores upon disability acquisition compared with individuals living in affordable housing.^33^ This suggests that living in unaffordable housing is likely to have a negative impact on mental health outcomes in people with disability; however, a definitive conclusion cannot be made from the results of a single study. Nonetheless, this tentative conclusion aligns with existing literature on the impact of unaffordable housing on health in the wider population, demonstrating similar findings of a negative effect on mental health outcomes, including from other Australian studies that used HILDA Survey data.^37^^,^^38^

### Strengths and limitations of this review

This review has a number of strengths. First, we adopted a rigorous and systematic approach as specified by JBI, including the use of 2 independent reviewers at each stage of screening and data extraction. Second, broadening our conceptualization of health outcomes to include a defined set of social determinants of health increased the likelihood of capturing additional studies. This expansion of our inclusion criteria also aligns with the broader focus on public health outcomes. Third, we only included studies in which disability was conceptualized in line with the ICF framework of disability, ensuring a focus on functional ability and participation, while avoiding the inclusion of medical conditions or diagnoses that fall outside of the scope of this review. Fourth, limiting the search to only peer-reviewed literature increased the likely quality of the included studies.

Despite these strengths, this review has several limitations. First, the exclusion of gray literature meant relevant studies published outside of academic journals may have been overlooked, including those from disability advocacy groups, research institutes, and not-for-profit organizations. This is particularly notable given that only 3 peer-reviewed studies were included. Second, this review may be affected by publication bias because important negative or inconclusive findings are less likely to be published than findings with a positive association.^39^ Relying solely on peer-reviewed literature also increases the risk of publication bias, because negative or inconclusive findings are more likely to be published in the gray literature.^39^ However, the impact of publication bias is reduced in a scoping review, where focus is not on critically appraising studies to answer a specific research question.^28^ Third, it is possible that we missed and failed to include eligible studies despite using comprehensive keywords to search multiple databases and manually searching the reference lists of the included studies, including studies that focused on specific health conditions or impairments associated with disability. However, the search strategy was developed in consultation with an experienced public health librarian to minimize this risk. Fourth, we may have missed studies by excluding those published prior to 2004 or those in a language other than English. Narrowing the time frame reduced the scope of our search, but it was necessary to exclude studies published before the introduction of the ICF model of disability, to ensure a consistent and current approach to conceptualizing disability. Even with the potential limitations of our search, it is unlikely that enough relevant studies were missed that would change our conclusions about the limitations of the existing research or the critical gap in research that was identified.

### Recommendations for future research and policy

Based on the limited evidence available on how housing affordability affects health in people with disability, we are unable to make any direct policy recommendations. However, the existing literature does indicate that living in unaffordable housing may have a negative impact on a broad range of health outcomes in the general population, particularly mental health. Given that people with disability are disproportionately exposed to unaffordable housing, and given the increasing evidence of the relationship between affordability and health in the wider population, it is reasonable to hypothesize that this relationship may at least be similar, and potentially more pronounced, in this priority population. Policies aimed at improving housing affordability for all are needed and have potential to benefit people with disability due to their greater exposure. Access to adequate and affordable housing is essential not only for health and well-being but also as a fundamental right under Article 28 of the Convention on the Rights of Persons with Disabilities.^1^

That only 3 studies were found in this review is an important finding in and of itself, starkly highlighting an absence of epidemiological evidence on this topic and the need for high-quality research to inform clear and targeted policy that can guide effective intervention. This can be achieved using targeted qualitative studies that allow researchers to capture in-depth perspectives of people with disability on their experiences with unaffordable housing and how it affects their health. Additionally, longitudinal, quantitative studies with large sample sizes representative of the broad population of people with disability are needed to generate robust estimates of how unaffordable housing affects various health outcomes for people with disability. Regardless of study design, future research must address the significant heterogeneity in the existing literature that this review demonstrates. Researchers must adopt a definition of disability in alignment with the internationally recognized ICF model of disability. Future studies should include broad populations of people with disability, encompassing a wide range of disability types and experiences, to produce more generalizable findings and facilitate the synthesis of results across studies. Research focused on specific disability subgroups is also necessary to provide critical insights into the unique experiences of these populations, acknowledging the diversity of the population of people with disability. Additionally, although mental health is likely the most sensitive outcome to housing affordability stress, studies should also pay attention to the numerous pathways through which a shortage of affordable housing can influence population health, including the trade-offs among affordability, suitability, quality, and location that can determine exposure to unhealthy home environments and influence people’s access to health-promoting resources such as services, social networks, employment, and greenspace. Beyond affordability, research is also needed to examine health impacts of other dimensions of housing for people with disability, including homelessness, tenure, and quality (including exposure to cold, damp, and mold), alongside further research on the physical and mental health effects of home modification and improved accessibility.^40^^-^^42^

Future research must also be committed to realizing the human rights of people with disability as stipulated in the United Nations Convention on the Rights of Persons with Disabilities. At a basic level, research must avoid an ableist approach that aims to “fix” people with disability.^43^ Wherever possible, studies should be co-created with and for people with disability, including genuine collaboration across the entire research project. This is more likely to produce ethical, beneficial research that avoids the perpetuation of harmful assumptions and stigma. The studies included in this review did not report whether they were co-created with people with disability. Notably, Semeah et al.^35^ adapted their written questionnaire format to allow participants with diverse disability types to participate, such as those who were blind or missing a limb. This highlights the importance of ensuring future research is accessible to meet the specific needs of participants, allowing for a diverse representation of disability. The results of future research should also be disseminated back to the disability community in an accessible format, including open-access publication.

## Conclusions

In this scoping review, we explored the impact of housing affordability on health outcomes in people with disability. The small number of eligible studies reveals a significant gap in the literature. Despite the small sample, all the studies provided evidence suggesting that living in unaffordable housing negatively affects mental health outcomes for people with disability. However, the high level of conceptual and methodological heterogeneity limited the ability to synthesize the findings and draw more definitive conclusions, and quantitative evidence regarding physical health outcomes was almost entirely lacking.

Understanding and addressing the impact of housing affordability on people with disability is a pressing public health priority. Affordable and appropriate housing is essential for health and well-being, and the historical exclusion of this population from research leaves significant gaps in the evidence base. The escalating housing affordability crisis in many countries increases the urgency of this issue, because people with disability are already at disproportionate risk of financial hardship and poor health outcomes. We found that although the very small body of existing research does support the importance of housing affordability for mental health in this population, there is very little research evidence available to reliably inform policy responses or intervention design. Future research must prioritize this population, using consistent definitions of disability and housing affordability, and clear outcome measures to assess a range of health outcomes. This will guide evidence-based policies and interventions that promote equitable access to affordable housing and improve health outcomes in this priority population.

## Supplementary Material

Web_Material_mxag004

## Data Availability

This study is a scoping review and did not involve the generation or analysis of new data. All relevant data are available in the source publications included in the review.
