# Supplementary material for: Housing affordability and health in people with disability: A scoping review
Source: Epidemiol Rev. 2026 Mar 9;48(1):mxag004. doi: 10.1093/epirev/mxag004 (PMC13082871; doi:10.1093/epirev/mxag004)
Supplement: Web_Material_mxag004 [file web_material_mxag004.pdf]

SUPPLEMENTARY MATERIAL

Housing affordability and health in people with disability: a scoping review

Kate Mason, Tanya Durrand, Glenda M. Bishop, Alex Sully, Zoe Aitken

Included materials:

Appendix S1: Search strategy

Appendix S1: Search strategy

All searches were limited to peer-reviewed publications in English, 2004 - 2024

Web Of Science (SSCI; SCI-EXPANDED):

TS=(disab* OR handicap* OR disabilit*) AND (TS=("affordable housing" OR "housing affordability" OR "unaffordable housing" OR "housing costs" OR "housing debt" OR "housing stress") OR TS=("rent* assistance" OR "housing assistance" OR "public housing" OR "social housing" OR "subsidised housing" OR "housing vouchers" OR "housing subsidies" OR "housing welfare" OR "housing benefit") OR TS=(("housing" OR "residential facilit*" OR "residence*" OR "living condition*" OR "apartment*" OR "condominium*" OR "house" OR "houses" OR "rental" OR "renting" OR "rent" OR "rents") NEAR/10 ("affordab*" OR "unaffordab*" OR "cost" OR "costs" OR "economics" OR "economic" OR "assistance" OR "subsidy" OR "subsidised" OR "subsidies" OR "voucher" OR "vouchers" OR "debt" OR "debts" OR "rent" OR "rents" OR "renting" OR "rental" OR "facilitat*" OR "loan" OR "loans" OR "bank" OR "banking" OR "banks" OR "credit" OR "finance" OR "financial" OR "secure" OR "insecure" OR "poor" OR "poverty")))

MEDLINE (Ovid):

((exp disabled person/ or (disab* or handicap* or disabilit*).mp.) and (affordable housing or housing affordability or unaffordable housing or housing costs or housing debt or housing stress).mp.) or ((exp disabled person/ or (disab* or handicap* or disabilit*).mp.) and (rent* assistance or housing assistance or public housing or social housing or subsidised housing or housing vouchers or housing subsidies or housing welfare or housing benefit).mp.) or ((exp disabled person/ or (disab* or handicap* or disabilit*).mp.) and (housing or residential facilit* or residence* or living condition* or apartment* or condominium* or house or houses or rental or renting or rent or rents).ti,kf. and (affordab* or unaffordab* or cost or costs or economics or economic or assistance or subsidy or subsidised or subsidies or voucher or vouchers or debt or debts or rent or rents or renting or rental or facilitat* or loan or loans or bank or banking or banks or credit or finance or financial or secure or insecure or poor or poverty).mp.)

SocIndex:

((((DE "PEOPLE with disabilities")) OR ((disab* OR handicap* OR disabilit*))) AND (affordable housing OR housing affordability OR unaffordable housing OR housing costs OR housing debt OR housing stress)) OR ((((DE "PEOPLE with disabilities")) OR ((disab* OR handicap* OR disabilit*))) AND (rent* assistance OR housing assistance OR public housing OR social housing OR subsidised housing OR housing vouchers OR housing subsidies OR housing welfare OR housing benefit)) OR (((((DE "PEOPLE with disabilities")) OR ((disab* OR handicap* OR disabilit*))) AND (housing OR residential facilit* OR residence* OR living condition* OR apartment* OR condominium* OR house OR houses OR rental OR renting OR rent OR rents)) AND (affordab* OR unaffordab* OR cost OR costs OR economics OR economic OR assistance OR subsidy OR subsidised OR subsidies OR voucher OR vouchers OR debt OR debts OR rent OR rents OR renting OR rental OR facilitat* OR loan OR loans OR bank OR banking OR banks OR credit OR finance OR financial OR secure OR insecure OR poor OR poverty))
